# Immunohistochemical Expression of AQP2 and HSP70 in Broiler Kidney Tissue Treated with *Salix tetrasperma* Roxb. Extract under Heat Exposure

**DOI:** 10.1155/2021/8711286

**Published:** 2021-10-18

**Authors:** Sugito Sugito, Etriwati Etriwati, Muslim Akmal, Erdiansyah Rahmi, Mira Delima, Zainal Abidin Muchlisin, Denny Irmawati Hasan

**Affiliations:** ^1^Faculty of Veterinary Medicine, Universitas Syiah Kuala, Banda Aceh 23111, Indonesia; ^2^Faculty of Agriculture, Universitas Syiah Kuala, Banda Aceh 23111, Indonesia; ^3^Faculty of Marine and Fisheries, Universitas Syiah Kuala, Banda Aceh 23111, Indonesia

## Abstract

The administration of plant extracts to broilers may be a way to mitigate the effects of heat stress. The importance of AQP2 and HSP70 compounds in maintaining the homeostasis of the chicken body when it is subjected to heat stress is well established. This study aims to determine the effect of giving the ethanolic extract of the leaves of *Salix tetrasperma* Roxb. on the immunohistochemical expression of AQP2 and HSP70 in exposed and unexposed broiler kidney tissue. This study used 36 samples of 28-day-old chicken kidneys. Chickens were kept in individual cages, provided with feed and drinking water *ad libitum*. The design used was a completely randomized design with 6 treatments and 6 replications: (a) chickens were reared in conditions exposed to heat (HS + 0); (b) chickens were reared in conditions exposed to heat and given *Salix* extract at a dose of 50 mg/L drinking water (HS + 50); (c) chickens were reared under heat-exposed conditions and given *Salix* extract at a dose of 100 mg/L drinking water (HS + 100); (d) chickens were reared in conditions without exposure to heat (n-HS + 0); (e) chickens were reared in conditions without exposure to heat and given *Salix* extract at a dose of 50 mg/L drinking water (nHS + 50); and (f) chickens were reared in conditions exposed without exposure to heat and given 100 mg/L drinking water (nHS + 100) of *Salix* extract. *Salix* extract was given for 24 hours and was renewed every 6 hours. The results showed that giving *Salix* extract 100 mg/L in drinking water to chickens exposed to heat (HS + 100) reduced the value of the H/L ratio. Giving *Salix* extract 50–100 mg/L in drinking water caused an upregulated AQP2 expression; on the other hand, it downregulated HSP-70 expression, in chicken kidney tubules both exposed to heat stress and nonexposed to heat stress. In conclusion, exposure to heat stress in broiler chickens and giving *Salix* extract can increase the formation of aquaporin 2 compounds and suppress the formation of HSP70.

## 1. Introduction

Chickens lose a lot of water via respiration and increase urine formation when they are subjected to heat stress. This water loss is the main mechanism used by broilers for thermoregulation [1, 2]. Under these conditions, kidney function will minimize the loss of dissolved sodium (Na) and increase tubular water reabsorption to help broilers reduce metabolic heat load [3]. The water reabsorption process in chicken kidney tubules involves aquaporin protein [4]. Aquaporin is a selective membrane channel protein expressed in various epithelial and endothelial cells of fluid transporting tissues [5, 6], including those of the gastrointestinal tract, kidney, and lungs which are required for active regulation of water transport [6, 7]. In cell membranes, aquaporins act as special pores/channels to pass water molecules. Thus, water can flow into and out of cells. The presence of this aquaporin compound allows water to move faster when compared to just going through the common osmosis process [7, 8].

Among the aquaporin (AQP) isoforms that have known roles in kidney tissue is aquaporin 2 (AQP2). AQP2 in the renal tubules plays a role in concentrating and regulating urine production to maintain body fluid homeostasis [9–11]. The avian kidneys express homologous aquaporins 1, 2, and 4, which have similar homology to aquaporins in mammals, but their distribution and function may not be the same [7, 9]. AQP2 is expressed along the cortical and medullary collecting ducts and responsible for the flow of water into and out of collecting duct epithelial cells [4, 12]. Several bioactive compounds of plant origin, such as polyphenols, flavonoids, and terpenoids, have been identified that can modulate the formation of aquaporin proteins [13–15].

When chickens experience heat stress, there is an increase in body temperature (hyperthermia) that might cause protein folding disorders [16, 17]. Therefore, the role of heat shock protein (HSP) compounds is essential. HSP compounds can act as molecular chaperones and function as shields for certain proteins through intracellular membranes [8, 17]. The presence of HSP compounds when there is an increase in heat stress is the body's effort to protect proteins that are sensitive to temperature changes [18, 19]. One of the HSPs that plays an important role when chickens experience heat stress is HSP70. Expression of the HSP70 molecule is found in almost all body tissues to protect cell damage from heat stress by maintaining denatured protein folds and preventing cellular apoptosis [19]. HSP70 has a cytoprotective role during heat stress exposure [17, 20]. There is a strong correlation between chicken genotype and HSP70 genotype with the ability of chickens to withstand heat exposure [21]. Chickens that produce higher HSP70 will be more tolerant when exposed to heat stress [22]. Heat stress is able to not only increase the expression of HSP70 but also the production of reactive oxygen species (ROS). There is an increase in HSP70 expression when chickens are exposed to heat, and this causes decreased ROS production [19, 20].

Previous studies have proven that giving *Salix tetrasperma* Roxb. extract to broilers is able to improve some of the negative effects of heat stress [23–27]. The improvement in this condition was due to the work activity of bioactive compounds contained in the *Salix* plant extract, which stimulated the activity of several biochemical compounds in the chicken body to reduce the negative effects of heat stress. The administration of *Salix* extract can stimulate the expression of antioxidant enzymes and prevent the formation of oxidative stress [28, 29]. Phytochemical test results from various parts of the *Salix tetrasperma* plant detected the content of various secondary bioactive compounds such as catechol, salicin and their derivatives, flavonoids, phenolics, terpenoids, saponins, and steroids [28, 30, 31]. It is suspected that the administration of *Salix* extract can affect the formation of AQP2 and HSP70 compounds. Thus, this study aims to examine the effect of giving *Salix* leaf extract on the expression of AQP2 and HSP70 molecules in the kidneys of broiler chickens exposed to chronic heat stress compared to those not exposed to heat stress.

## 2. Materials and Methods

### 2.1. Experimental Animals

This study used 36 samples of 28-day-old broiler chicken kidneys. Before being given treatment, the chickens were adapted and randomly placed in individual cages from the age of 15 days to the age of 20 days. Individual cages measuring 40 × 30 × 40 cm were equipped with places to eat and drink. Feed and drinking water were provided throughout the day (*ad libitum*). The chicken feed used was commercial feed (CP Vivo 512) with the following nutritional compositions: crude protein 20.5%, crude fat 8.0%, crude fiber 6%, calcium 0.9%, phosphorus 0.6%, and 3.050 kcal ME/kg.

### 2.2. Animal Ethics Approval

This study was supervised and obtained ethical approval for the use of experimental animals numbered 52/KEPH/IV/2019 from the Ethics Commission for the Use of Experimental Animals, Faculty of Veterinary Medicine, Universitas Syiah Kuala.

### 2.3. Experimental Design

A completely randomized design was used in this study consisting of 6 treatments and 6 replications. The treatments tested were as follows: (a) chickens were reared in heat-exposed conditions (HS + 0), (b) chickens were reared in heat-exposed conditions and given *Salix* extract at a dose of 50 mg/L in drinking water (HS + 50), (c) chickens were reared under heat-exposed conditions and given a *Salix* extract dose of 100 mg/L in drinking water (HS + 100), (d) chickens were reared in conditions without exposure to heat (n-HS + 0), (e) chickens were reared in conditions without exposure to heat and given *Salix* extract at a dose of 50 mg/L in drinking water (nHS + 50), and (f) chickens were reared in conditions without exposure to heat and given *Salix* extract at a dose of 100 mg/L in drinking water (nHS + 100). Each treatment consisted of 6 replications.

The treatment was given since the chicken was 21 days old. Heat exposure was given 4 hours per day for 7 consecutive days and applied by increasing the room temperature of the cage to a temperature of 34 ± 1°C and relative humidity (RH) at 75–80%. *Salix* extract was administered throughout (*ad libitum*) for 7 days and renewed every 6 hours. Chickens reared without exposure to heat stress were kept in cages with a temperature of 25 ± 1°C and RH at 50 ± 55%. The temperature and humidity were controlled using an air conditioner (AC). On day 28, all chickens were sacrificed for kidney and blood collections.

### 2.4. Heterophile/Lymphocyte (H/L) Ratio Calculation

The ratio of heterophile/lymphocyte (H/L) was determined from 2 peripheral blood smear preparations per chicken blood sample. A thin layer of blood was applied to the surface of the glass slide and then dried. The slides were fixed in 95% ethanol for 2 minutes and stained with Wright's stain solution. A total of 100 leukocyte differential cells were counted for each slide (total 100 cells per chicken) using a binocular microscope (Olympus BX51) with a magnification of 400 times. The heterophile and lymphocyte ratio was calculated by dividing the number of heterophiles with the number of lymphocytes.

### 2.5. Immunohistochemical of Kidney Sample Preparation

Immunohistochemical of sample preparation in this study referred to [32]. Kidney samples were cut into 1 cm × 1 cm × 0.5 cm and washed by flowing tap water. Kidney organ sections were fixed in 10% neutral buffered formalin for 24 hours at room temperature, transferred to ethanol, and then, embedded in paraffin. The kidney tissue was cut to a thickness of 5 m and processed for immunohistochemical labeling. Renal tissue was heparinized in xylene and rehydrated through a series of graded ethanol at room temperature. This was followed by immersing the tissue in distilled water for 20 minutes at room temperature and washing it three times with 1x phosphate-buffered saline (1xPBS).

The antigen retrieval process was carried out by boiling the tissues in a buffered citrate solution at the temperature of 100°C for 15 minutes. The tissue preparation was then removed and left for 20 minutes at room temperature and then washed 3 times with 1xPBS. Endogenous peroxidase activity was removed by incubating tissues in hydrogen peroxidase solution for 20 minutes and washed 3 times with 1xPBS. Protein blocking was applied for 20 minutes and washed once with 1xPBS. Tissues were then incubated with the HSP70 antibody (Anti-HSP70 antibody (BB70) ab53496, Abcam; 1 : 200 in 1xPBS) or AQP2 antibody (Product ID ABIN5688893 antibodies online GmbH; 1 : 200 in 1xPBS) and further incubated at the temperature of 5°C overnight. The tissue was then washed three times in 1xPBS. The tissues were then incubated in Biotinylate Goat Anti-Polyvalent for 15 minutes at room temperature, washed three times with 1xPBS, and then, incubated with Streptavidin peroxidase for 15 minutes and washed three times with 1xPBS. Next, tissues were incubated with DAB + chromogen (1 drop: 1.5 ml) for 3 minutes and washed with running water for 10 minutes and washed 3 times with 1xPBS at room temperature. After applying Mayer's hematoxylin as a counterstain, tissues were then dehydrated with graded ethanol and cleaned with xylene. All washing stages were carried out 5 minutes each, whereas ethanolic dehydration steps were carried out 3–5 minutes each.

### 2.6. AQP2 Expression and HSP70 Score Calculation

The results of AQP2 and HSP70 immunohistochemical staining were evaluated using a binocular microscope (Olympus BX51) at 200x magnification. The following procedure was used to assess the expression of AQP2 and HSP70 in a semiquantitative manner: AQP2 and HSP70 staining intensity was categorized into four classifications (intensity scores): 0 (negative, no staining), 1 (weak, light brown), 2 (moderate, brown), and 3 (strong, dark brown). The percentages that were positive were sorted into five groups (percentage scores): 0 (0%), 1 (1–25%), 2 (26–50%), 3 (51–75%, and 4 (76–100%). Positive staining was determined by the formula overall score = percentage score × intensity score. The resulting staining score was used as the final staining score for AQP2 and HSP70 (0–12). The optimal limit value is 4, and a score of ≥4 is considered as a high AQP2 and HSP70 expression, while a score of <4 is considered as a low AQP2 and HSP70 expression.

### 2.7. Data Analysis

The H/L ratio data were analyzed using analysis of variance (ANOVA). If from the results of this analysis, there was a difference in the average treatment, it might be further analyzed using Duncan's comparative test. To see the comparison of aquaporin and HSP70 expression (quantified), the Kruskal–Wallis test was used, followed by pairwise comparison.

## 3. Results

### 3.1. Heterophile and Lymphocyte (H/L) Ratio

**Figure 1:**
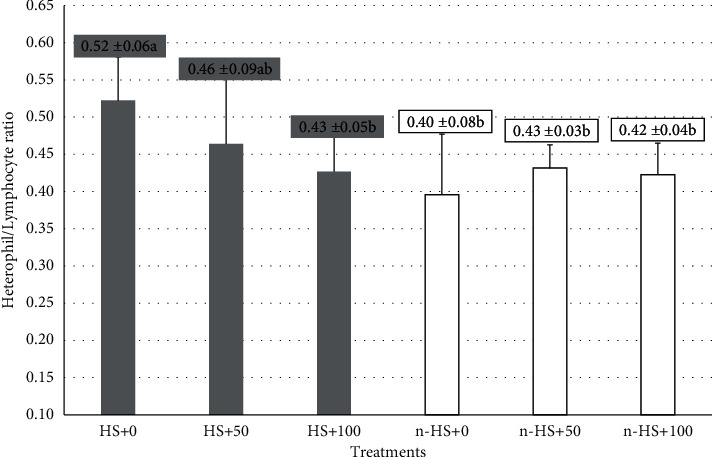
The average values of the heterophile/lymphocyte ratio of chicken blood *Salix* leaf extract in underexposed and unheated conditions. Different superscript letters on the graph showed significant differences (*P* < 0.05).

The average value of the H/L ratio in chickens that were not exposed to heat stress and followed by the administration of *Salix* extract is presented in Figure 1. Administration of *Salix* leaf extract at a dose of 100 mg per liter (mg/L) in drinking water of broilers aged 28 days exposed to heat stress (a temperature of 34 ± 1°C and RH of 75–80%) caused a significant decrease (*P* < 0.05) in the value of the H/L ratio (Figure 1). The highest H/L ratio (0.52 ± 0.06) was found in the treatment (a), where chickens were exposed to heat stress and not given *Salix* extract. Meanwhile, the lowest ratio (0.40 ± 0.08) was obtained in the treatment (d), where chickens were not exposed to heat stress and were not given *Salix* extract. The results of Duncan's comparative test showed that chickens exposed to heat stress and given the *Salix* extract showed an effect if given a dose of 100 mg/L drinking water. However, the administration of *Salix* extract to broilers that were not exposed to heat stress only showed a difference in the average value of the H/L ratio with treatment (a).

### 3.2. Aquaporin 2 (AQP2) Expression

**Figure 2:**
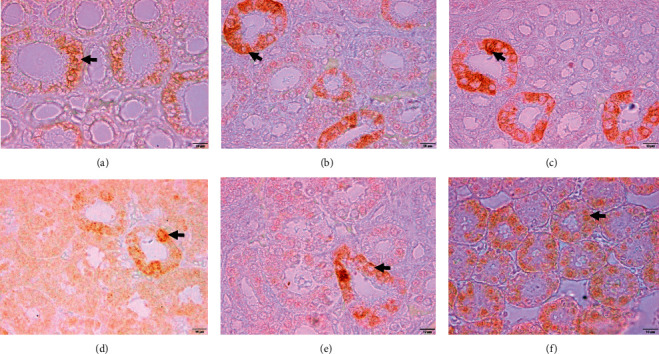
The results of the staining of aquaporin 2 on tissue in chicken kidneys. (a) HS + 0, AQP2 positive in tubule cells (light brown). (b) HS + 50, AQP2 positive in tubule cells (brown). (c) HS + 100, AQP2 positive in tubule cells (dark brown). (d) nHS + 0, AQP2 positive in tubule cells (light brown). (e) nHS + 50, AQP2 positive in tubule cells (light brown). (f) nHS + 100, AQP2 positive in tubule cells (light brown).

**Figure 3:**
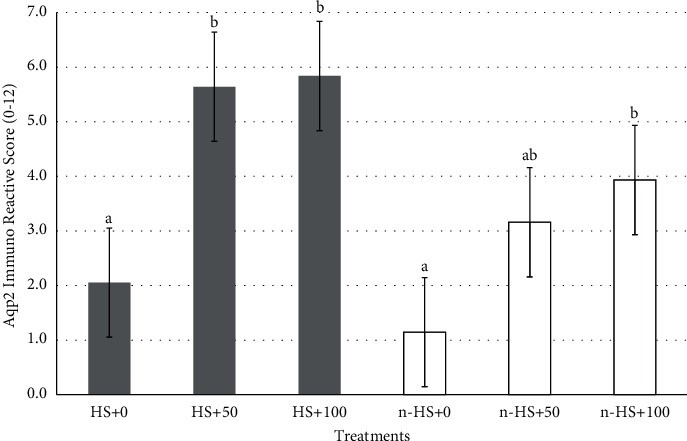
The average score of aquaporin 2 protein staining results in chicken kidney tubule tissue with *Salix* leaf extract under heat-exposed and unheated conditions. Different letters indicate significant (*P* < 0.05) differences among treatments.

The results of the IHC staining showed that AQP2 was located on the apical membrane of the renal collecting tubule (Figure 2). The average score of AQP2 immunoreactive cell expression is presented in Figure 3. The statistical test results indicate that the administration of *Salix* extract increases the AQP2 expression score in kidney tissue, both in chickens exposed to heat stress and those not exposed to heat stress. Further test results showed that giving *Salix* extract at doses of 50 and 100 mg/L of drinking water to chickens exposed to heat (HS + 50 and HS + 100 treatments) could significantly increase (*P* < 0.05) AQP2 expression score. However, if given to chickens that were not exposed to heat stress, the effect was seen when given at a dose of 100 mg/L of drinking water (treatment nHS + 100) (Figure 3). The results of this study also showed that heat exposure did not affect (*P* > 0.05) the AQP2 expression score in chicken kidney tissue (HS + 0 treatment) with the average score not significantly different from the treatment of chickens that were not exposed to heat stress (n-HS + 0 treatment).

### 3.3. Expression of Heat Shock Protein-70 (HSP70)

**Figure 4:**
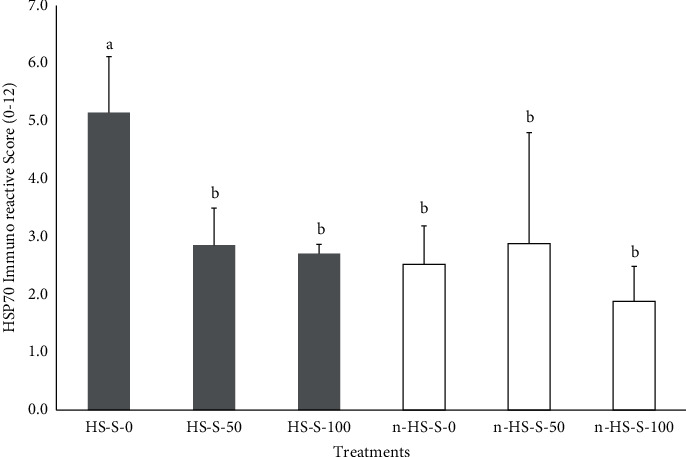
The average score of HSP70 protein staining on chicken kidney tubule tissue that was given *Salix* leaf extract under heat-exposed and non-heat-exposed conditions. Different superscript letters on the graph showed significant differences (*P* < 0.05).

**Figure 5:**
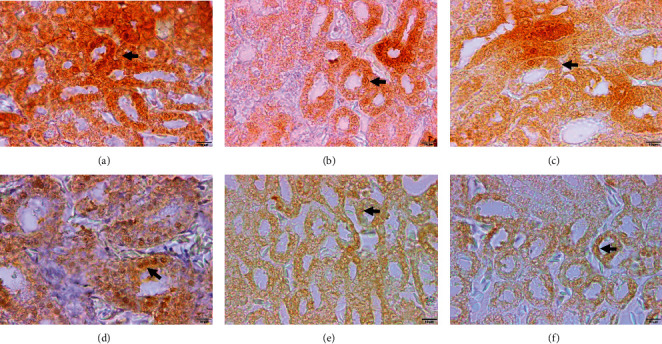
Results of HSP70 protein staining on tissue in chicken kidneys on a scale of 10 mm. (a) HS + 0, HSP70 positive in tubule cells (dark brown). (b) HS + 50, HSP70 positive in tubule cells (brown). (c) HS + 100, HSP70 positive in tubule cells (light brown). (d) nHS + 0, HSP70 positive in tubule cells (brown). (e) nHS + 50, HSP70 positive in tubule cells (light brown). (f) nHS + 100, HSP70 positive in tubule cells (light brown).

Scores of HSP70 immunohistochemical staining results in chicken kidneys for each treatment are presented in Figure 4. The location of the presence of HSP70 was detected in tubular epithelial cells and renal glomerular endothelial cells (Figure 5). Statistical test results showed that heat stress exposure significantly (*P* < 0.05) increased HSP70 expression. Furthermore, the administration of *Salix* extract in broiler chickens exposed to heat stress significantly decreased (*P* < 0.05) renal HSP70 expression. On the other hand, the administration of *Salix* extract to broiler chickens not exposed to heat stress did not show any change in the average score of HSP70 immunoreactive cells (Figure 4).

## 4. Discussion

Measurement of the H/L ratio is an accurate and effective indicator to measure stress levels in chickens exposed to chronically hot environments. An increase in the value of the H/L ratio indicates the chicken is in a state of stress. The increase in the number of heterophile cells and the decrease in chicken lymphocyte cells were caused by the increased secretion of glucocorticoid and cortisol hormones when chickens were subjected to heat stress [33–35]. The results of the measurement of the H/L ratio in this study showed that giving *Salix* leaf extract to chickens exposed to chronic heat stress showed an effect when given at a dose of 100 mg/L. The results of this study support several previous studies that giving plant extracts to chickens experiencing heat stress can reduce the value of the H/L ratio as a sign of the reduced impact of chronic heat stress [36]. The reduced impact of heat stress is due to the role of bioactive compounds contained in plant extracts in reducing reactive oxygen species (ROS) and increasing antioxidant activity [37]. The decrease in the H/L ratio in this study was expected to be due to the activity of the phytochemical content of the *Salix* plant leaf extract. In *Salix* plants, there are bioactive compounds that act as antioxidants, including derivatives of phenolic compounds and flavonoids [28, 30, 31, 38–40]. Various types of flavonoids and their derivatives are beneficial in birds experiencing chronic heat stress and become less effective when given to birds that are not exposed to heat [41]. Polyphenols are natural antioxidants that can reduce oxidative stress [37]. The dietary of *Salix alba* bark powder can improve the oxidative status of broiler chickens by increasing the antioxidant activity of antioxidant enzymes against oxidation [29].

The condition of heat exposure in broilers causes changes in kidney activity to minimize urine loss and solutes. Decreased glomerular filtration rate, solutes, and tubular Na reabsorption rate can help broilers reduce metabolic heat load [3]. Among various protein compounds, aquaporin compounds play an important role in reducing the effects of heat stress on chickens. The presence of aquaporins plays an essential role in maintaining water homeostasis in the body, especially in the process of urine formation and reducing body fluid loss [4]. Among the isoforms of aquaporin compounds that play an active role is aquaporin 2 (AQP2). AQP2 compounds function to regulate fluid reabsorption in the renal tubules [42]. AQP2 compounds are found in the collecting ducts and renal tubules. The presence of AQP2 in the kidney plays a role in water reabsorption [43]. Aquaporin 2 is a small protein located in the collecting tubules of the kidney and plays an important role in the concentration and production of urine [44]. The presence of AQP might affect the loss of body fluid of chickens because when broiler chickens experience heat stress, they might lose more water (through gasping and urination) and result in increased water consumption to compensate for water loss and to increase heat dissipation capacity. On the other hand, water retention is reduced due to increased electrolyte excretion in urine and feces [1].

The results of this study showed that, in chickens exposed to heat stress, there was an increase in AQP2 expression in the kidney tubules. This increase in AQP2 is expected to be related to dehydration due to heat exposure of chickens. Renal AQP2 expression decreases during overhydration and increases after dehydration [45]. The same thing happened in water-deprived (dehydrated) mice showing increased AQP2 expression [46]. In chickens, restriction of water intake might regulate AQP2 expression [47]. Arginine vasotocin (AVT) has an important role in urine concentration through stimulation of the pathway, gene expression, and AQP2 protein synthesis in the collecting duct [4].

The administration of *Salix* extract to chickens exposed to heat stress caused an increase in AQP2 expression that was more than two times higher than that of control chickens and relatively less when given to chickens that were not exposed to heat stress. The increase in AQP2 expression in the kidney tubules was triggered by the activation of bioactive compounds contained in *Salix* leaf extract and the stimulus for an increase in environmental temperature. *Salix* plant species are known to be rich in quercetin compounds [28]. Several polyphenolic compounds of plant origin, such as quercetin, play a role in modulating the expression of several aquaporin isoforms in body tissues [13, 14]. Quercetin can prevent and reverse the decrease in water permeability caused by oxidative stress [13]. In rats suffering from chronic kidney disease, quercetin administration can improve kidney function, reduce oxidative stress factors, and can reduce renal tubular damage [48].

Exposure to heat stress in broilers can cause an increase in the expression of HSP70 in the kidneys [49, 50]. The concentration of HSP70 expression can be an indicator of the level of stress experienced by an organism. High levels of HSP70 expression indicate high stress levels, and vice versa [21]. This increase in HSP70 expression is in response to the presence of heat stressors to increase cell survival and protect proteins from folding, disaggregation, and protein degradation. Increased formation of HSP70 protects cellular proteins from heat stress damage [51].

The results of this study indicate that heat exposure in broiler chickens causes an increase in the expression of HSP70 in the kidneys. This is in line as reported by several other studies that exposure to heat increases the amount of HSP70 protein expression in broiler kidney tissue [52, 53]. An increase in environmental temperature can trigger the activity of the heat shock protein transcription factor in the form of an increase in the synthesis of HSP70 mRNA, increasing the concentration of HSP70 [54]. The increase in medullary HSP70 mRNA was seen rapidly after hyperthermia and remained elevated for at least 48 hours [55]. Induction of HSP70 expression can protect cells from damage induced by apoptosis and from damage caused by oxidative injury, fibrosis, and inflammation [56]. In kidney tissue, HSP compounds are an important part of the intracellular defense system, which is activated by various types of cellular stress. The presence of HSPs in cells plays a role in stabilizing cell structure, increasing cell resistance to apoptosis and necrosis, and maintaining the potential for further regeneration [57].

The results of this study showed that giving *Salix* leaf extract to chickens exposed to heat stress could reduce HSP70 expression and had no effect on HSP70 expression when given to chickens that were not exposed to heat stress. This decrease in the expression of the HSP70 protein in the kidney causes its role as a renoprotective against tissue damage due to the stress response to be reduced. It has been reported that the administration of *Salix* plant ethanol extract to broilers suffering from heat stress did not show any protective effect on kidney tissue damage [24]. According to the work in [58], the administration of grape seed extract can reduce HSP70 gene expression in the broiler liver before and during chronic heat stress conditions.

The decrease in HSP70 expression is deemed to be due to the activity of one of the bioactive compounds contained in the *Salix* extract, namely, quercetin. Quercetin is an inhibitor of protein transcription and HSP70 expression [20, 59]. Quercetin compounds inhibit the expression of HSP70 through the mechanism of heat shock factor 1 (HSF1) [60]. The inhibition of HSP70 synthesis by quercetin is influenced by the temperature and type of stressor [61]. The results of this study proved that giving *Salix* extract to chickens that were not exposed to hot environmental conditions did not affect the expression of HSP70 in the kidneys. In hyperthermic conditions, the response to quercetin activity inhibits the synthesis and intracellular accumulation of HSP70 occurs in a complex manner [62].

The response of giving *Salix* extract on the expression of AQP2 and HSP70 in this study was very different. Moreover, there was no interaction between the expressions of the two compounds. The administration of *Salix* extract at a dose of 100 mg/L to broilers that were not exposed to heat stress did not affect the expression of HSP70. On the contrary, there was an increase in the expression of AQP2. The results of this study differ from those previously reported, which reported an interaction between AQP2 and HSP70 compounds in tissues. The interaction of AQP2 with HSP70 plays a role in the intracellular transport of AQP2 and the degradation or recycling of AQP2 [63]. CHIP (carboxyl terminal of HSP70-interacting protein) is known to be a major regulator of AQP2 degradation through HSP70 [64]. It is suspected that the activity of the bioactive compounds contained in the *Salix* extract requires interaction with other compounds produced by the chicken body during heat exposure.

## 5. Conclusions

There is an increase in the formation of AQP2 and HSP70 in the kidney tubules of chickens exposed to heat stress. The administration of *Salix* leaf extract could increase the expression of AQP2, in the kidney tubules of chickens both exposed to heat stress and not exposed to heat stress, on the other hand, suppressing the expression of HSP70, in the condition of chickens both exposed to and not exposed to heat stress.

## Data Availability

The data used to support the findings of this study are available from the corresponding author upon request.
